# Capsule Production and Glucose Metabolism Dictate Fitness during *Serratia marcescens* Bacteremia

**DOI:** 10.1128/mBio.00740-17

**Published:** 2017-05-23

**Authors:** Mark T. Anderson, Lindsay A. Mitchell, Lili Zhao, Harry L. T. Mobley

**Affiliations:** a Department of Microbiology and Immunology, University of Michigan Medical School, Ann Arbor, Michigan, USA; b Biostatistics Department, University of Michigan School of Public Health, Ann Arbor, Michigan, USA; University of Washington

## Abstract

*Serratia marcescens* is an opportunistic pathogen that causes a range of human infections, including bacteremia, keratitis, wound infections, and urinary tract infections. Compared to other members of the *Enterobacteriaceae* family, the genetic factors that facilitate *Serratia* proliferation within the mammalian host are less well defined. An *in vivo* screen of transposon insertion mutants identified 212 *S. marcescens* fitness genes that contribute to bacterial survival in a murine model of bloodstream infection. Among those identified, 11 genes were located within an 18-gene cluster encoding predicted extracellular polysaccharide biosynthesis proteins. A mutation in the *wzx* gene contained within this locus conferred a loss of fitness in competition infections with the wild-type strain and a reduction in extracellular uronic acids correlating with capsule loss. A second gene, *pgm*, encoding a phosphoglucomutase exhibited similar capsule-deficient phenotypes, linking central glucose metabolism with capsule production and fitness of *Serratia* during mammalian infection. Further evidence of the importance of central metabolism was obtained with a *pfkA* glycolytic mutant that demonstrated reduced replication in human serum and during murine infection. An MgtB magnesium transporter homolog was also among the fitness factors identified, and an *S. marcescens mgtB* mutant exhibited decreased growth in defined medium containing low concentrations of magnesium and was outcompeted ~10-fold by wild-type bacteria in mice. Together, these newly identified genes provide a more complete understanding of the specific requirements for *S. marcescens* survival in the mammalian host and provide a framework for further investigation of the means by which *S. marcescens* causes opportunistic infections.

## IMPORTANCE

*Serratia marcescens* is a remarkably prolific organism that replicates in diverse environments, including as an opportunistic pathogen in human bacteremia. The genetic requirements for *S. marcescens* survival in the mammalian bloodstream were defined in this work by transposon insertion sequencing. In total, 212 genes that contribute to bacterial fitness were identified. When sorted via biological function, two of the major fitness categories identified herein were genes encoding capsule polysaccharide biogenesis functions and genes involved in glucose utilization. Further investigation determined that certain glucose metabolism fitness genes are also important for the generation of extracellular polysaccharides. Together, these results identify critical biological processes that allow *S. marcescens* to colonize the mammalian bloodstream.

## INTRODUCTION

*Serratia marcescens* is a member of the *Enterobacteriaceae* family that is found ubiquitously in the environment. This species is known to be associated with plants, animals, insects, freshwater, and soil and is also recognized as an opportunistic pathogen of humans (1). As an opportunistic pathogen, *S. marcescens* causes a wide range of both health care-associated and community-acquired infections, including bacteremia, keratitis, wound infections, and urinary tract infections (1). The diverse environments in which this organism thrives suggest a remarkable physiologic flexibility. Recent analysis of the *S. marcescens* pangenome has demonstrated that a large number of poorly conserved accessory gene elements exist between isolates of this species, suggesting a genetic basis by which *S. marcescens* can occupy diverse niches (2). Furthermore, a comparison of the gene contents of an insect isolate and a human isolate of *S. marcescens* demonstrated a large degree of genetic diversity (3). This physiologic flexibility and genetic diversity underscore the need to assess the requirements for bacterial fitness of specific isolates under conditions that closely model their niche of origin. Of particular interest to this work is the ability of *S. marcescens* to survive during mammalian bloodstream infection. Bacteremia caused by *S. marcescens* is a serious disease associated with a mortality rate of 23 to 52% (4–6).

Previous work to define genes required for *S. marcescens* survival during infection has focused primarily on individual gene targets or genetic screens using invertebrate hosts. One virulence factor that has emerged from these studies is the ShlA hemolysin, which has been demonstrated to have cytolytic activity against multiple eukaryotic cell types and contributes to pathogenesis in a murine model of pneumonia (7–10). Other *S. marcescens* proteins that are proposed virulence factors include secreted proteases (11–13), the PhlA extracellular phospholipase (14), and iron acquisition proteins (15). Two studies have also assessed *in vivo* fitness with *Caenorhabditis elegans* as a host to identify *S. marcescens* transposon mutants that exhibit decreased nematode killing. By using a derivative of insect isolate Db10 (16), Kurz et al. identified 23 transposon mutants that were attenuated in the nematode model (10). In similar work, Iguchi et al. also identified 12 transposon mutants of Db10 that were attenuated in *C. elegans*, corresponding to eight genetic loci not previously identified in the Kurz screen (3). This same study identified a putative polysaccharide capsule biosynthesis locus and determined that the *wza* gene within this locus contributed to *C. elegans* infection.

Polysaccharide capsules are produced by multiple *Enterobacteriaceae* species and are important mediators of immune resistance by interfering with opsonophagocytosis and complement-mediated killing (17, 18). *S. marcescens* is known to produce a capsule consisting of acidic polysaccharides, the specific composition of which varies between isolates (19, 20). Despite the potential importance of *S. marcescens* capsule during mammalian bloodstream infection, the genetic basis of capsule biosynthesis remains understudied in this organism.

To further define *S. marcescens* genes required for *in vivo* bacterial fitness, specifically within a mammalian host, we conducted a transposon insertion sequencing (INSeq) screen with a bank of ~32,000 mutants in a murine model of bacteremia. We report herein the identification of 212 genes, encompassing a wide range of predicted functions that contribute to *S. marcescens in vivo* fitness. Two of the major biological processes identified in this work are capsule biosynthesis and glucose metabolism functions.

## RESULTS

### Establishment of a murine model of *S. marcescens* bloodstream infection.

Eleven *S. marcescens* bloodstream infection isolates were obtained from the University of Michigan Health System for this study, and the genome sequence of each isolate was determined. From this collection, strain UMH9 was selected for further characterization on the basis of the ability to reproducibly colonize mice at a high level and an antibiotic susceptibility profile that was compatible with available genetic manipulation tools (see Fig. S1 in the supplemental material). Strain UMH9 is a nonpigmented isolate that exhibits intermediate resistance (32 µg/ml) to the clinically relevant piperacillin-tazobactam drug combination.

10.1128/mBio.00740-17.6 FIG S1  Relative resistance of *S. marcescens* isolates to various antibiotics. *S. marcescens* clinical isolates were cultured at 37°C in LB medium containing the antibiotics indicated, and bacterial growth was measured by determining the OD_600_. The resistance of each strain to a given antibiotic was calculated relative to that of untreated cultures. The dotted line indicates equivalent growth in the presence and absence of antibiotic. Results represent the mean of triplicate cultures ± the standard deviation. Antibiotics were used at the following concentrations: ampicillin, 100 µg/ml; zeocin, 50 µg/ml; kanamycin, 50 µg/ml; chloramphenicol, 30 µg/ml; tetracycline, 15 µg/ml. Download FIG S1, EPS file, 0.6 MB. Copyright © 2017 Anderson et al. 2017 Anderson et al. This content is distributed under the terms of the Creative Commons Attribution 4.0 International license.

**FIG 1:**
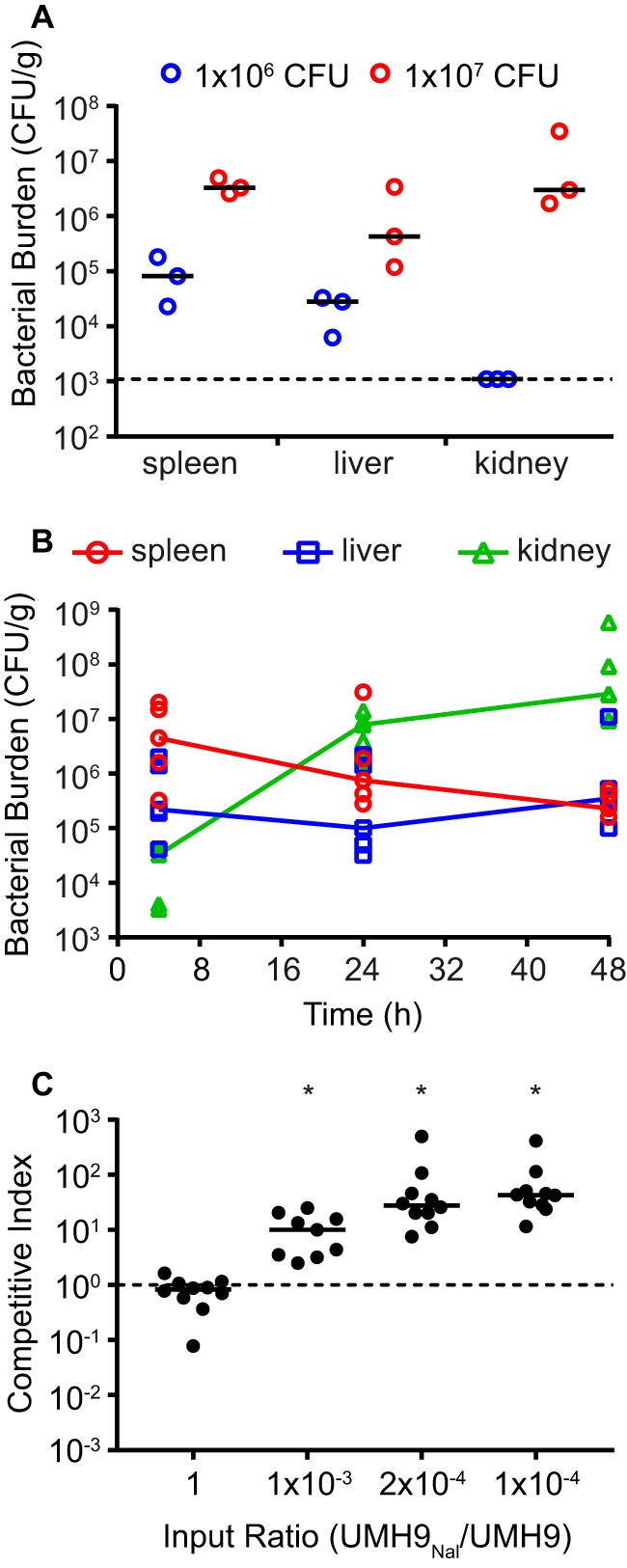
Establishment of a murine model of *S. marcescens* bloodstream infection. (A) C57BL/6 mice were inoculated with *S. marcescens* strain UMH9 via tail vein injection at the doses indicated. Mice were sacrificed at 24 h postinfection, and the number of viable bacteria in each organ was determined. The limit of detection is indicated by the dotted line. (B) Mice were inoculated as described for panel A with 1 × 10^7^ CFU of strain UMH9, and the bacterial burden was monitored over time. Lines indicate the median bacterial burden in each organ. (C) Evaluation of colonization bottlenecks in the spleen with a spontaneously resistant strain (UMH9^Nal^) during coinfection with the UMH9 parent. Mixed inocula, at the ratios indicated, were used to infect mice with a total of 1 × 10^7^ CFU. After 24 h, mice were sacrificed and the CI was determined by measurement of viable counts. A CI of 1.0 (dotted line) indicates a lack of fitness advantage for either strain. CI values that differed significantly from a hypothesized median value of 1.0, as determined by a Wilcoxon signed-rank test (*P* < 0.01), are indicated with asterisks.

The overall objective of this work was to define the bacterial genes necessary for survival in the mammalian bloodstream; therefore, a murine model of bacteremia was established for strain UMH9. Infection of C57BL/6 mice with 1 × 10^7^ CFU via tail vein injection resulted in robust colonization of the spleen, liver, and kidneys at 24 h (Fig. 1A). In contrast, inoculation with 1 × 10^6^ CFU resulted in a low overall bacterial burden and mice infected with 1 × 10^8^ CFU were moribund prior to 24 h (data not shown). The 1 × 10^7^ CFU dose was used for all subsequent experiments. The numbers of colonizing bacteria in the spleens, livers, and kidneys of infected mice were determined 4 h postinfection to assess the proportion of the inoculum that was capable of establishing infection. The greatest density of bacteria was found in the spleen at this early time point, indicating a decreased likelihood of colonization bottlenecks at that site (Fig. 1B). Although UMH9 replicates to high levels in the murine kidney for ≥48 h, the low initial bacterial density in this organ was considered problematic with regard to the potential loss of individual mutants from a large inoculum.

To further assess the potential for colonization bottlenecks, a spontaneous nalidixic acid-resistant mutant was isolated and used in coinfections with parent strain UMH9. Mice were infected with different ratios of the two strains, with strain UMH9^Nal^ representing any given clone in a mixed population of transposon insertion mutants. If colonization was limited by bottleneck effects, UMH9^Nal^ would be expected to exhibit stochastic loss from the population and a competitive disadvantage (competitive index [CI] of <1.0). At input ratios as low as 1 × 10^−4^ (UMH9^Nal^ to UMH9), no spontaneous loss of the resistant strain was observed from the spleen-colonizing population (Fig. 1C). Since an *in vitro* outgrowth step was necessary to recover the low proportion of UMH9^Nal^ from spleen homogenates, the observation that UMH9^Nal^ seemingly outcompeted the parent strain *in vivo* may be due to a marginal growth advantage of strain UMH9^Nal^ (see Fig. S2A). In support of this conclusion, a 1:1 ratio of the two strains that was plated directly from spleen homogenates exhibited a neutral CI (~1.0) (see Fig. S2B). Together, these data demonstrate that the UMH9 bacteremia infection model can accommodate a high population diversity without the stochastic loss of individual clones due to bottlenecks during colonization.

10.1128/mBio.00740-17.7 FIG S2  Relative fitness of UMH9 and UMH9^Nal^ *in vitro* and assessment of colonization bottlenecks in the spleen. (A) Strains UMH9^Nal^ and UMH9 were cocultured in LB medium at ratios of 1:100 (circles) and 1:1,000 (squares), and the CI over time was determined by viable count. None of the CI values were significantly different from the hypothesized value of 1.0 (dotted line), except for the 1:100 culture at 6 h postinoculation (asterisk), as determined by one-sample *t* test (*P* > 0.01). (B) Assessment of infection bottlenecks by competition infections with a 1:1 mixed inoculum of strains UMH9^Nal^ and UMH9. The CI was determined by direct plating of spleen homogenates (closed circles) and after outgrowth of spleen homogenates in LB medium (open circles). There was no significant difference between the median CI values determined by the two methods, as determined by the Mann-Whitney test (*P* > 0.01). The median CI values from both methods were also not significantly different from the hypothesized value of 1.0 by the Wilcoxon signed-rank test (*P* > 0.01). Download FIG S2, EPS file, 0.6 MB. Copyright © 2017 Anderson et al. 2017 Anderson et al. This content is distributed under the terms of the Creative Commons Attribution 4.0 International license.

### Transposon insertion sequencing screen for *in vivo* fitness genes.

**FIG 2:**
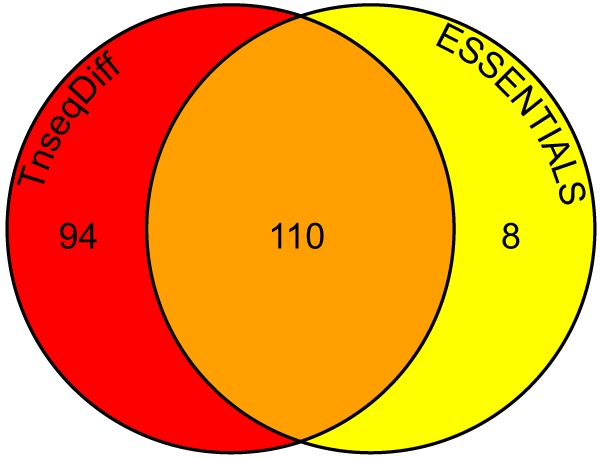
Numbers of INSeq fitness genes determined by TnseqDiff and ESSENTIALS analyses. The number of UMH9 genes having statistically significant (adjusted *P* < 0.05) fitness defects of ≥2-fold was determined by the TnseqDiff (red) and ESSENTIALS (yellow) programs. The number of fitness genes identified by both methods is in the orange area.

A *mariner*-based transposon carried by suicide plasmid pSAM-Cm (21) was used to generate a random library of transposon insertion mutants in strain UMH9 (see Fig. S3). Five pools containing equivalent numbers (~6,400) of mutants were used to infect four mice per pool. Spleens were collected from infected mice after 24 h. The insertion sites from input and output pools were PCR amplified and sequenced via Illumina to determine the relative abundance of each transposon mutant (22). The ESSENTIALS pipeline (23) was used to map sequence reads and identify essential and conditionally essential (fitness) genes. A newly developed R package called TnseqDiff, which uses insertion level information to identify fitness genes, was also applied to the data (L. Zhao, M. T. Anderson, W. Wu, Y. Li, H. L. T. Mobley, and M. A. Bachman, submitted for publication). In total, the library consisted of ~32,000 unique insertions (~25,000 in open reading frames [ORFs]), and the two methods combined identified 212 UMH9 transposon-disrupted genes that exhibited a ≥2 (range, 2.9 to 154.3)-fold decrease (adjusted *P* < 0.05) in bacterial fitness (see Data Set S1). The majority of the fitness genes were identified by both methods, although the TnseqDiff package identified a larger number of fitness genes that did not meet the significance criteria of ESSENTIALS (Fig. 2). Sequence reads from the input population were also used to predict that 712 genes in the UMH9 genome are essential (adjusted *P* < 0.05) (23). Complete essential scores (see Data Set S2) and conditionally essential scores (see Data Set S3) for each gene, as well as the genomic locations of all transposon insertions (see Data Set S4) can be found in the supplemental material.

10.1128/mBio.00740-17.8 FIG S3  Random insertion of transposons into the *S. marcescens* genome. Purified genomic DNA from strain UMH9 (parent) or randomly isolated transposon insertion mutants was digested with SalI. A digoxigenin-labeled DNA fragment corresponding to an internal portion of the kanamycin resistance gene of the *mariner* transposon was used in a Southern blot assay to detect the integration of the transposon. DNA from plasmid pSAM-Cam was used as a positive control (+). Download FIG S3, EPS file, 2.8 MB. Copyright © 2017 Anderson et al. 2017 Anderson et al. This content is distributed under the terms of the Creative Commons Attribution 4.0 International license.

10.1128/mBio.00740-17.2 DATA SET S1  *S. marcescens* genes with significant fitness defects (≥2.0-fold change; adjusted *P* < 0.05) as determined by ESSENTIALS and the TnseqDiff package. Download DATA SET S1, XLSX file, 0.1 MB. Copyright © 2017 Anderson et al. 2017 Anderson et al. This content is distributed under the terms of the Creative Commons Attribution 4.0 International license.

10.1128/mBio.00740-17.3 DATA SET S2  *S. marcescens* UMH9 essential gene scores determined by the ESSENTIALS pipeline. Download DATA SET S2, XLSX file, 0.5 MB. Copyright © 2017 Anderson et al. 2017 Anderson et al. This content is distributed under the terms of the Creative Commons Attribution 4.0 International license.

10.1128/mBio.00740-17.4 DATA SET S3  Conditionally essential gene scores for all *S. marcescens* UMH9 genes determined by ESSENTIALS and the TnseqDiff package. Download DATA SET S3, XLSX file, 1 MB. Copyright © 2017 Anderson et al. 2017 Anderson et al. This content is distributed under the terms of the Creative Commons Attribution 4.0 International license.

10.1128/mBio.00740-17.5 DATA SET S4  Genomic locations of transposon insertions for all five *S. marcescens* UMH9 mutant pools. Download DATA SET S4, XLSX file, 6.7 MB. Copyright © 2017 Anderson et al. 2017 Anderson et al. This content is distributed under the terms of the Creative Commons Attribution 4.0 International license.

**FIG 3:**
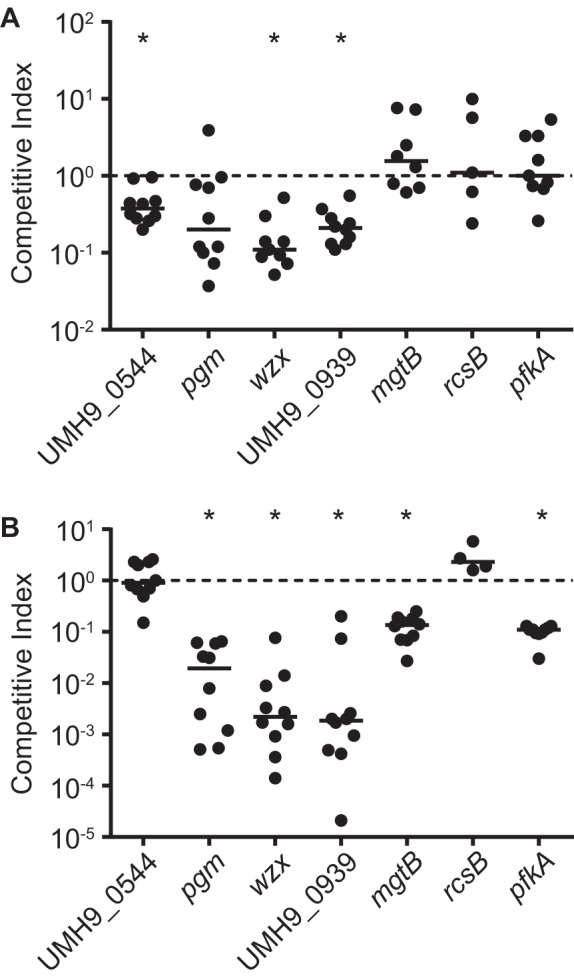
Validation of fitness gene mutants by competition infection. Mixtures (1:1) of the UMH9 parent strain and strains harboring mutations in the genes indicated were used to coinfect mice via tail vein injection. The numbers of viable bacteria present in the spleen (A) and kidneys (B) were determined and used to calculate the CI. A CI of 1.0 is indicated by the dotted line. Asterisks indicate mutants that exhibited a significant CI decrease from the hypothesized median value of 1.0 as determined by Wilcoxon signed-rank test (*n* = 10, *P* < 0.01).

Seven fitness genes identified by both the TnseqDiff package and ESSENTIALS were chosen for further study and for validation of the INSeq screen. Deletion-insertion mutations were constructed for each of the genes described in Table 1, and the resulting strains were tested for *in vivo* fitness defects in competition with the UMH9 parent strain by using the murine bacteremia model. Three of the mutants tested exhibited significant fitness defects in the spleen, and five were outcompeted in the murine kidney (CI of <1) (Fig. 3). The results of these experiments validated the findings of the genetic screen and confirmed that six of the seven genes tested contribute to *S. marcescens* fitness in the mammalian host. Importantly, none of the seven mutants exhibited a general growth defect when cultured *in vitro* (see Fig. S4). Of the strains tested, only the *rcsB* mutant failed to exhibit a significant fitness defect in either the spleen or the kidneys. The genes listed in Table 1 are predicted to encode a wide range of biological functions; therefore, subsequent *in vitro* assays were designed to explore the role of each gene product during *S. marcescens* infection.

10.1128/mBio.00740-17.9 FIG S4  *In vitro* growth of *S. marcescens* fitness gene mutants. The growth of all of the fitness gene mutants generated in this study was compared to that of wild-type strain UMH9. Bacteria were cultured in LB medium, and the OD of each culture was determined every 15 min. Results are reported as the mean of triplicate cultures ± the standard deviation. Download FIG S4, EPS file, 0.9 MB. Copyright © 2017 Anderson et al. 2017 Anderson et al. This content is distributed under the terms of the Creative Commons Attribution 4.0 International license.

**TABLE 1:** Selected *S. marcescens* fitness genes

Gene or locus tag	Predicted function	INSeq fitness defect, FC (adjusted *P* value)^a^	
		ESSENTIALS^b^	TnseqDiff^c^
UMH9_0544	Hypothetical protein	19.1 (<0.001)	19.2 (<0.001)
*pgm*	Phosphoglucomutase	27.8 (<0.001)	9.2 (<0.001)
*wzx*	Membrane flippase for polysaccharide translocation	9.5 (<0.001)	8.6 (<0.001)
UMH9_0939	β-1,3-Glucosyltransferase	87.2 (<0.001)	21.3 (<0.001)
*mgtB*	Magnesium transport ATPase, P type	153.1 (<0.001)	26.3 (<0.001)
*rcsB*	Capsular biosynthesis response regulator	16.6 (<0.001)	29.0 (<0.001)
*pfkA*	Phosphofructokinase	18.3 (<0.001)	9.5 (<0.001)

^a^ The inverse of the output/input ratio is reported as the fold change (FC) with the adjusted *P* value.

^b^ Level of fitness defect as determined by the ESSENTIALS pipeline.

^c^ Level of fitness defect as determined with the TnseqDiff function in the TnseqDiff package.

### *S. marcescens* capsule is a major fitness determinant.

**FIG 4:**
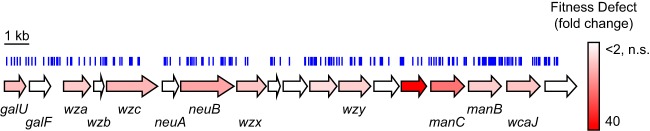
Involvement of the *S. marcescens* capsule biosynthesis locus in bacterial fitness. The ORFs of 18 genes within the putative capsule biosynthesis locus of strain UMH9 are depicted as arrows. Arrows without gene designations represent genes that encode hypothetical proteins. The level of fitness defect associated with each gene as determined by INSeq (ESSENTIALS) is indicated by red shading. Genes with a fitness defect of <2-fold or a fitness defect that was not statistically significant (n.s., adjusted *P* ≥ 0.05) are indicated by open arrows. The locations of 191 individual transposon insertions identified within this region are represented by blue vertical lines.

In competition infections with the wild-type strain, the *wzx* mutant exhibited 9- and 450-fold losses of fitness in the spleen and kidney, respectively (Fig. 3). Wzx flippase proteins are best studied with regard to their role in inner membrane translocation of lipid-linked O-antigen repeat units (24, 25); however, Wzx homologs also function in sugar translocation with *wzy*-dependent capsule synthesis systems (18). The UMH9 *wzx* gene is located within an 18-gene locus with similarity to predicted capsule biosynthesis loci from other *S. marcescens* isolates (3). The hypothesized function of this genetic region in capsule production, rather than O-antigen synthesis, is reinforced by the presence of *wza*, *wzb*, and *wzc* outer membrane capsule transport gene homologs (18). A total of 191 unique transposon insertions were identified in the 22-kb capsule locus, and in addition to *wzx*, 10 other genes were determined by INSeq to contribute to bacterial fitness, underscoring the critical importance of this genetic region for *S. marcescens* infection (Fig. 4). Further analysis of the capsule locus among the 11 different clinical isolates sequenced in this study demonstrated a high degree of heterogeneity, consistent with the known variation of *S. marcescens* capsule composition and structure (see Fig. S5) (20). Fitness genes outside this region with predicted roles in capsule biosynthesis were also identified; notably, *rfaH* transposon mutants exhibited an ~9-fold fitness defect. The RfaH antitermination factor is known to control the transcription of multiple extracytoplasmic component genetic loci in other bacterial species, including the group 1 capsule genes of *Escherichia coli* (26, 27).

10.1128/mBio.00740-17.10 FIG S5  Multisequence alignment of *S. marcescens* capsule biosynthesis loci. (A) The capsule genetic loci of 11 *S. marcescens* bloodstream infection isolates were aligned by the Clustal Omega algorithm. Each sequence fragment spans the region between the *galU* gene encoding a UTP–glucose-1-phosphate uridylyltransferase and the *lipB* gene encoding the ATP-binding component of an ABC exporter, the latter of which is not involved in capsule biosynthesis. Green segments represent conserved sequences. (B) Neighbor-joining tree generated from the alignment in panel A with the uncorrected pairwise distance of each sequence. Download FIG S5, EPS file, 1.1 MB. Copyright © 2017 Anderson et al. 2017 Anderson et al. This content is distributed under the terms of the Creative Commons Attribution 4.0 International license.

**FIG 5:**
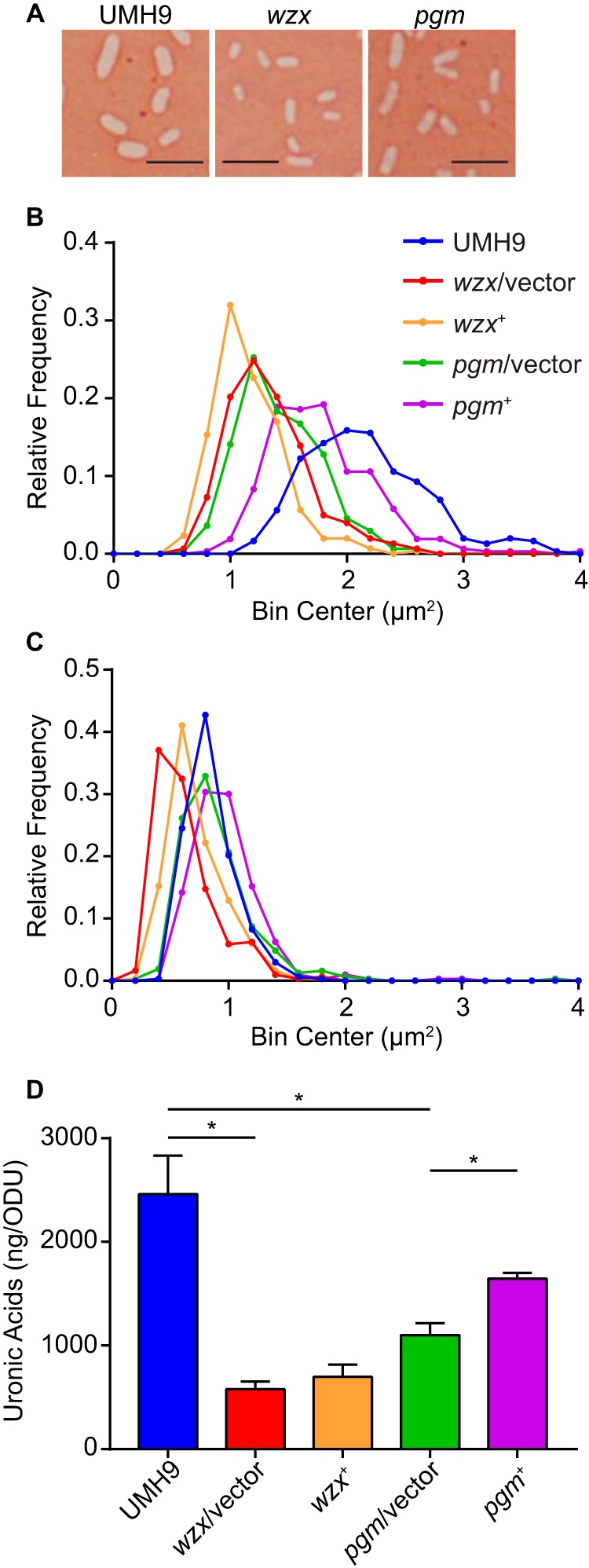
Detection of *S. marcescens* capsule by negative staining and measurement of uronic acids. (A) Assessment of capsule production by *S. marcescens* strains by CR negative staining. Scale bars, 5 µm. (B) Distribution of CR negative-stained area from individual cells of the *S. marcescens* strains indicated. (C) Distribution of crystal violet-stained area of individual *S. marcescens* cells. Colors correspond to the same strains that are indicated in panel B. Approximately 300 cells of each strain were measured for panels B and C. (D) Bacteria from overnight cultures of *S. marcescens* were used to measure the level of extracellular uronic acids by colorimetric assay. The amount of uronic acids present in each sample was normalized to the OD of each culture and was calculated on the basis of a standard curve generated with glucuronic acid. Asterisks indicate uronic acid levels that different significantly between strains, as determined by *t* test (*P* < 0.01, *n* = 3). ODU, OD unit.

To assess capsule production, *S. marcescens* strains were stained with Congo red (CR) dye and visualized by light microscopy. Wild-type strain UMH9 produced a robust negative-stain area surrounding bacterial cells when cultured at physiologic temperatures, indicative of capsule production (Fig. 5A). This result is in contrast to that of the *wzx* mutant, which appeared to yield a smaller negative-stain area. Quantitative analysis demonstrated that wild-type bacteria produced a median negative-stain area of 2.10 µm^2^ compared to 1.28 µm^2^ for the *wzx* mutant harboring the empty vector (Mann-Whitney test *P* < 0.0001), indicating a defect in capsule production (Fig. 5B). Attempts to complement the *wzx* mutation by providing *wzx* on a multicopy plasmid (*wzx*^+^) failed to restore capsule production in this assay, a result that may be due to the polarity of the *wzx* mutation on transcriptionally linked capsule-associated genes (Fig. 4). The area of crystal violet-stained *S. marcescens* strains were also measured as a control, the results of which demonstrate a more similar overall distribution between strains compared to CR (Fig. 5C). As expected, the largest difference between CR and crystal violet stain areas was observed with the fully encapsulated wild-type strain.

Uronic acids are carboxylated sugars that can be found in the capsular polysaccharides of certain Gram-negative bacterial species, including *E. coli* strains with group 1 capsules and *Klebsiella pneumoniae* (18, 28). Previous work to characterize the capsule serotypes of *S. marcescens* identified glucuronic acid and galacturonic acid as common components of acid polysaccharide fractions from several different reference strains (19, 20). As a further assessment of capsule production in UMH9, extracellular uronic acids from the relevant *S. marcescens* strains were quantitated. A significant reduction in uronic acid levels was observed in the *wzx*/vector strain compared to those of the wild type, reinforcing the lack of capsule production in this mutant (Fig. 5D). As observed with the CR stain assay, genetic complementation of the *wzx* mutation was unable to restore uronic acid production.

### The importance of glucose metabolism during infection.

**FIG 6:**
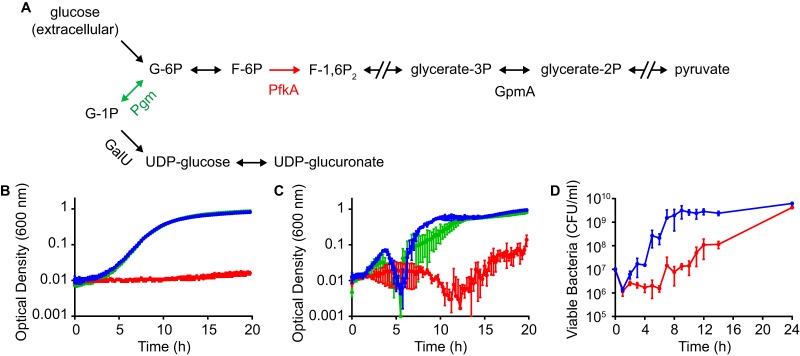
*S. marcescens* fitness genes involved in glucose metabolism and growth in serum. (A) Predicted enzymatic pathways for the conversion of glucose into pyruvate and UDP-glucuronate by *S. marcescens*. Gene products that contribute to *S. marcescens* fitness during bloodstream infection as determined by INSeq are indicated. Broken arrows represent multiple steps in product and substrate interconversion that are not shown. Abbreviations: G-1P, glucose-1-phosphate; G-6P, glucose-6-phosphate; F-6P, fructose-6-phosphate; F-1,6P_2_, fructose-1,6-bisphosphate. (B) Growth of *S. marcescens* strains in M9 medium supplemented with 0.4% glucose as the sole carbon source. (C) Growth of *S. marcescens* strains in M9 medium supplemented with 20% HI normal human serum. (D) Growth of *S. marcescens* in M9 with serum as determined by viable counts. Reported values are the mean of triplicate cultures ± the standard deviation. The *S. marcescens* strains tested were wild-type UMH9 (blue) and *pfkA* (red) and *pgm* (green) mutants.

Niche-specific acquisition and utilization of nutrients are critical aspects of bacterial physiology during infection. Four *S. marcescens* gene products predicted to function in the enzymatic manipulation of glucose were identified as fitness factors by INSeq. PfkA catalyzes the first committed step in glycolysis by converting fructose-6P into fructose-1,6P_2_, whereas GpmA catalyzes the interconversion of glycerate-3P and glycerate-2P further downstream in the pathway (Fig. 6A). A third gene, *pgm*, encodes phosphoglucomutase, which promotes the bidirectional conversion of glucose-6P to glucose-1P and is not involved in the conversion of extracellular glucose into pyruvate. Lastly, the *galU* gene product catalyzes the conversion of glucose-1P into the nucleotide sugar UDP-glucose (29). The UMH9 *galU* gene is located within the capsule biosynthesis locus and exhibited a 10-fold fitness defect by INSeq (Fig. 4). While the loss of GalU function is expected to disrupt capsule biosynthesis in these bacteria, *S. marcescens galU* mutants are likely pleiotropic because of the central role of UDP-glucose in bacterial metabolism.

Since loss of *gpmA* or *pfkA* is likely to result in the same phenotype with regard to the utilization of glucose as a sole carbon source, initial efforts were focused on characterization of just the *pfkA* null mutant. Assessment of growth in M9 medium containing glucose demonstrated that *pfkA* was required for glucose utilization, as expected (Fig. 6B). In contrast, the *pgm* mutant exhibited no growth defect under the same conditions. Since *pfkA* was identified as a fitness factor in a bloodstream infection model, it was hypothesized that a functional glycolysis pathway would be necessary for growth of *S. marcescens* in serum. M9 medium containing 20% heat-inactivated (HI) human serum promoted the robust growth of both the wild-type UMH9 strain and the *pgm* mutant; however, *S. marcescens* lacking *pfkA* showed a severe growth defect under these conditions (Fig. 6C). *S. marcescens* cultured in the presence of serum formed macroscopic aggregates during exponential growth (data not shown), resulting in a decrease in optical density (OD) during this phase. To confirm that the loss of OD was not due to decreased viability, the CFU counts of UMH9 and the *pfkA* mutant in serum culture were determined by colony counting. The results of this experiment confirmed the requirement for *pfkA* in the presence of serum and showed no loss of viability of the wild-type strain (Fig. 6D). Interestingly, after 24 h of culture, the *pfkA* mutant achieved the same number of viable bacteria as UMH9, suggesting that while glucose may serve as an *S. marcescens* carbon source in serum, it is likely that alternative carbon sources may also be utilized. Together with the INSeq analysis findings, these results indicate that glycolysis is a critical pathway during *S. marcescens* bloodstream infection.

**FIG 7:**
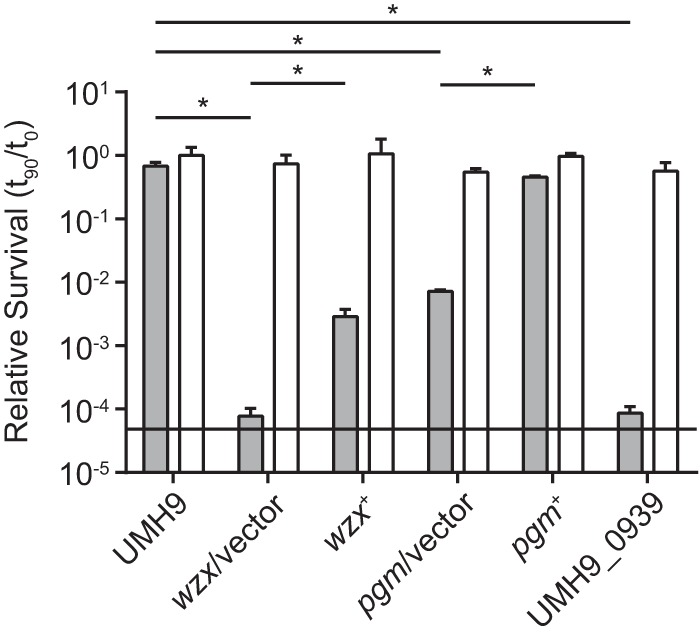
Identification of *S. marcescens* genes required for resistance to serum bactericidal activity. The relative survival of *S. marcescens* strains in PBS containing 10% normal human serum (gray bars) and HI serum (open bars) was determined. The mean number of viable bacteria present after a 90-min incubation at 37°C relative to time zero (± the standard deviation) is reported for triplicate suspensions. Each asterisk indicates a significant difference in the survival of two strains in 10% normal human serum as determined by *t* test (*P* < 0.01, *n* = 3). The horizontal line indicates the limit of detection.

The minimal growth defect observed with the *pgm* mutant in both M9 containing glucose and M9 with human serum (Fig. 6B and C) suggested that glucose-1P was not a major glycolytic substrate under these conditions. To address the contribution of *pgm* to *S. marcescens* fitness, the relative survival of bacteria incubated in the presence of 10% normal human serum and HI human serum was tested. Wild-type bacteria were largely resistant to the bactericidal activity of human serum under these conditions, exhibiting a minimal loss of viability over a 90-min incubation (Fig. 7). In contrast, the number of viable *pgm* mutant bacteria harboring the empty vector decreased by >2 orders of magnitude after exposure to serum, with serum resistance restored by genetic complementation of the *pgm* mutation (*pgm*^+^). Treatment with HI serum resulted in minimal changes in bacterial viability over the course of the experiment, indicating that the loss of *pgm* mutant viability was likely due to complement-mediated bactericidal activity. On the basis of the observed serum sensitivity of the *pgm* mutant and the predicted function of Pgm in the production of nucleotide sugar precursors (Fig. 6A), it was hypothesized that the enhanced susceptibility of the *pgm* mutant to complement components was due to altered production of cell surface polysaccharides. Therefore, mutations that disrupt capsule or lipopolysaccharide (LPS) biogenesis functions would be predicted to have a similar phenotype. The UMH9_0939 ORF is predicted to encode a β-1,3-glucosyltransferase and is located within the putative O-antigen biosynthetic gene cluster (3). Mutation of UMH9_0939 resulted in a robust fitness defect (Fig. 3) and conferred an even greater loss of serum resistance than the *pgm* mutation (Fig. 7). The *wzx* mutant strain also exhibited a complete loss of serum resistance, and surprisingly, partial restoration of viability was observed upon genetic complementation. The reason for the discrepancy in the complementation phenotypes of the *wzx* mutant is unclear; however, we speculate that the level of capsular polysaccharide required for detection by the CR stain and uronic acid measurement may differ from that required for serum resistance. Together, these results demonstrate that surface polysaccharides are required for *S. marcescens* serum resistance. To test more directly if *pgm* contributes to surface polysaccharide biogenesis, *pgm* mutant bacteria were assayed for loss of capsule. Indeed, the *pgm*/vector strain produced significantly less capsule than the wild type by CR staining (median = 1.37 µm^2^, *P* < 0.0001) (Fig. 5A and B) and had lower levels of extracellular uronic acids (Fig. 5D). Genetic complementation of the *pgm* mutation resulted in partial restoration of both the capsule and uronic acid phenotypes.

The UMH9_0544 gene is located ~1.9 kb upstream of the *pgm* ORF and is predicted to encode an uncharacterized protein containing a ribbon-helix-helix transcriptional repressor motif (InterPro accession no. IPR010985). Although mutation of UMH9_0544 resulted in an ~3-fold loss of fitness in the murine spleen (Fig. 3), no change in sensitivity to human serum was detected in comparison to wild-type bacteria (data not shown). As a result, the function of the UMH9_0544 gene product remains uncharacterized.

### A magnesium transport protein contributes to fitness.

**FIG 8:**
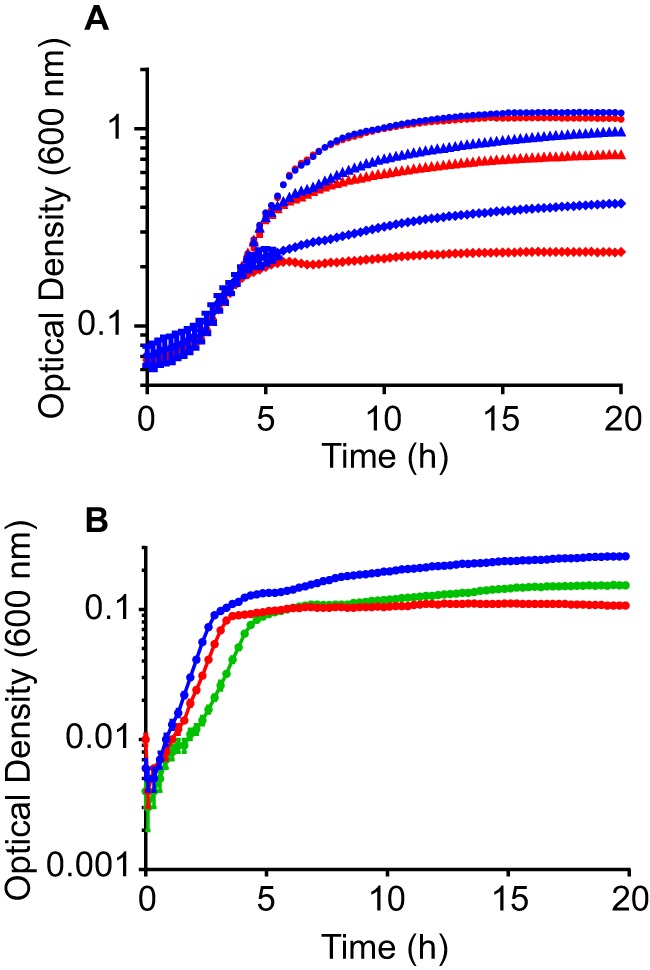
Growth of the *mgtB* mutant in various concentrations of magnesium. (A) *S. marcescens* UMH9 (blue) and *mgtB* mutant (red) strains were cultured in Chelex-treated M9 medium, and the growth of each strain was measured by determining the OD. Magnesium sulfate was added to cultures at the following concentrations: circles, 32 µM; triangles, 16 µM; diamonds, 4 µM. (B) Growth of the wild-type UMH9 (blue) and *mgtB*/vector (red) and *mgtB*^+^ (green) mutant strains was assayed in Chelex-treated M9 medium containing 4 µM magnesium sulfate. The mean ODs of triplicate cultures ± the standard deviations are shown.

Magnesium is a divalent cation that has multiple biological functions, including as an enzymatic cofactor and a neutralizer of negatively charged macromolecules. There are four known types of bacterial magnesium transporters (30). On the basis of sequence identity with characterized proteins from other species, the *S. marcescens* UMH9 genome encodes at least one homolog of each type, namely, CorA, MgtE, MgtA, and MgtB (data not shown). However, among the predicted UMH9 magnesium transporter genes, only the *mgtB* homolog was identified as a fitness factor during murine bloodstream infection (Table 1). Analysis of an *mgtB* deletion mutant during competition infections with the wild-type parent demonstrated a 7-fold fitness defect in murine kidneys, confirming the role of *mgtB* as an *S. marcescens* fitness gene (Fig. 3B). The function of UMH9 MgtB as a magnesium transporter was investigated by culturing bacteria in Chelex-treated M9 medium supplemented with different concentrations of magnesium. In the presence of low (4 µM) and intermediate (16 µM) concentrations of magnesium, the *mgtB* mutant grew to a lower density than the UMH9 parent strain (Fig. 8). Interestingly, a difference between the two strains was observed only in the postexponential phase. When bacteria were cultured in medium containing as little as 32 µM magnesium, there was a negligible growth difference between the *mgtB* mutant and wild-type strains. The reduction in bacterial density observed in the *mgtB* mutant strain in 4 µM magnesium was partially restored by providing *mgtB* on a multicopy plasmid (*mgtB*^+^) (Fig. 8B). Although the growth difference between the two strains in the presence of low concentrations of magnesium is consistent with a role in magnesium transport, the phenotype appears to be specific to the postexponential phase and was not observed in the presence of ≥32 µM magnesium. These observations suggest that additional transport mechanisms may compensate for the loss of MgtB at higher concentrations of magnesium and that MgtB is not required during rapid *S. marcescens* growth. It is also possible that MgtB functions most efficiently only under specific environmental conditions, since there was a clear difference in the requirement for *mgtB* in the spleen and in the kidneys during competition infections (Fig. 3).

## DISCUSSION

The global emergence of *Enterobacteriaceae* species harboring metallo-β-lactamase and serine β-lactamase genes that provide resistance to “last-resort” antibiotics of the carbapenem class underscores the urgency with which additional treatment options are needed to combat infections caused by these versatile pathogens (31, 32). The identification and characterization of genes that are required for bacterial survival *in vivo* provide a strategy through which novel targets for antimicrobial therapy may be identified. In this study, we identified *S. marcescens* genes that contributed significantly to bacterial fitness in the mouse using an INSeq-based genetic screen. Two previous *in vivo* genetic screens with an *S. marcescens* insect isolate were conducted in a nematode model, together resulting in the identification of 30 transposon insertion mutants that were attenuated for *C. elegans* killing (3, 10). Two common fitness pathways were identified by the nematode screen and our murine screen. An *mgtB* promoter mutation was shown to attenuate *C. elegans* killing but did not differ from the wild-type strain during infection of *Drosophila melanogaster* or a pulmonary epithelial cell line (10). The *wza* gene, encoding a capsule polysaccharide export protein, was also identified in a *C. elegans* screen (3) and this study, indicating that capsule production is important for infection of both invertebrate and vertebrate host species.

One gene notably absent from our INSeq results was *shlA*. The ShlA extracellular hemolysin (7) facilitates cytotoxic activity against cultured cell lines (8), and multiple studies have demonstrated a role for ShlA in pathogenesis (9, 10, 33). The absence of *shlA* identification in our study was not due to a lack of appropriate mutants, since multiple *shlA* transposon insertion mutants were detected in our library (data not shown). Rather, it is likely that *shlA* mutant bacteria in a larger population of hemolysin-producing organisms may benefit from the secreted hemolysin of neighboring bacteria, and thus, no lack of fitness would be detected.

The Rcs phosphorelay is a well-studied regulatory network that controls the expression of numerous genes and is conserved among the members of the family *Enterobacteriaceae* (34, 35). Colonic acid and group 1 capsules are mutually exclusive extracellular polysaccharides that are produced by different strains of *E. coli*, and the biosynthesis of each involves the transcriptional activation of different target genes by the RcsB response regulator (26, 36). The *S. marcescens rcsB* mutant did not exhibit enhanced sensitivity to serum bactericidal activity (data not shown) and did not have a significant fitness defect in competition infections (Fig. 3), inconsistent with a role in capsule production. However, 50% of the mice infected with a 1:1 mixture of wild-type and *rcsB* mutant bacteria succumbed to infection prior to 24 h. This is in contrast to mice infected with the wild-type strain alone (Fig. 1) or in combination with other fitness gene mutants (Fig. 3), which routinely survived to 24 h and beyond. These results suggest that the *rcsB* mutation increases the mouse mortality rate without altering the fitness of the bacterium. In *S. marcescens*, RcsB is known to repress *shlAB* expression both through the FlhDC regulatory pathway and by directly binding to the *shlAB* promoter (37). Therefore, the increased mortality rate of mice infected with *rcsB* mutants is potentially due to increased expression of the *shlAB* hemolysin-encoding locus. Consistent with this hypothesis, overexpression of *shlAB* through deletion of an independent two-component regulatory system, RssAB, also results in an increased murine mortality rate (33). The discrepancy between the identification of *rcsB* as an INSeq fitness gene and the lack of a fitness defect in competition infections with the wild-type strain remains to be determined.

In the case of the *mgtB* and *pfkA* genes, the fitness defect observed by INSeq in bacteria harvested from the spleen was not replicated in engineered mutants in competition with the wild type (*pgm* conferred a fitness defect that was not statistically significant) (Fig. 3A). One possible explanation for this result is that the INSeq experiment may be more sensitive to loss of fitness from any single mutant in the population, given that the ratio of each mutant (~1:6,000) is much lower than in the competition infections (1:1). It is also important to note that the *pgm*, *mgtB*, and *pfkA* mutants did exhibit significant fitness defects when bacteria from the kidneys were assayed (Fig. 3B). The reason for the organ-specific differences in the fitness defects of the *mgtB* and *pfkA* mutants remains untested. However, it was determined that wild-type *S. marcescens* colonizing the spleen gradually decrease in abundance over 48 h, in contrast to the prolific expansion of bacteria in the kidneys in the same time period (Fig. 1B), suggesting that the selective pressures in these two environments likely differ.

The *S. marcescens* UMH9 capsule biosynthesis locus shares genetic similarity with group 1 capsule and colonic acid biosynthesis loci of *E. coli* and certain capsule loci of *K. pneumoniae* (18, 28). A notable difference in comparison to the group 1 and *K. pneumoniae* capsules is the lack of a *wzi* gene within the *Serratia* capsule locus. Wzi is an outer membrane protein involved in tethering of the capsule polysaccharide to the bacterial surface, and the presence of *wzi* is one factor that differentiates cell-associated capsular polysaccharide loci from secreted exopolysaccharide loci, such as colonic acid (18, 38, 39). Interestingly, a predicted homolog (61% identity) of the *E. coli* B44 (group 1, K30) Wzi protein is located at a distal chromosomal locus in UMH9 and other *S. marcescens* isolates. Previous characterization of extracellular polysaccharides from several *S. marcescens* strains demonstrated that the O antigen of LPS and capsule polysaccharides are composed of neutral and acidic sugars, respectively (19, 20). The sugar acids glucuronic acid and galacturonic acid were commonly observed constituents of the different *S. marcescens* K serotypes tested but were lacking among different O-antigen serotypes (20). Therefore, the decrease in extracellular uronic acids observed in the *pgm* mutant strain (Fig. 8D) is likely specific to decreased or modified capsule production. This hypothesis is also consistent with the observed decrease in extracellular uronic acids in the *wzx* flippase mutant. However, it should be noted that because Pgm acts upstream in the pathway converting glucose-6P to the polysaccharide precursors UDP-glucose and UDP-glucuronate (Fig. 5A), it is possible that *pgm* mutation results in LPS defects in addition to those associated with the capsule.

In summary, this work represents a comprehensive mutational analysis of an *S. marcescens* clinical isolate to identify fitness genes required for mammalian infection. We have demonstrated the importance of capsule, glucose metabolism, and a magnesium transport protein by using this system and have identified many additional *S. marcescens* fitness genes that await functional analysis.

## MATERIALS AND METHODS

### Bacterial strains and culture conditions.

All of the *S. marcescens* isolates used in this study (see Text S1) originated from patient bloodstream infections and were collected by the University of Michigan Hospital System Clinical Microbiology Laboratory. Bacterial isolates were identified as *S. marcescens* as part of routine clinical laboratory procedures. The 16S rRNA locus from each isolate was also PCR amplified and sequenced (data not shown) by previously described methods (40). Strain UMH9^Nal^ was obtained by isolating a spontaneously resistant mutant of wild-type strain UMH9 following culture on LB agar containing 50 µg/ml nalidixic acid. LB medium (41) was used for the routine culture of *S. marcescens* and *E. coli* strains. Antibiotics were used at the following concentrations: kanamycin, 50 µg/ml; gentamicin, 10 μg/ml; ampicillin, 100 µg/ml; streptomycin, 100 µg/ml.

10.1128/mBio.00740-17.1 TEXT S1  Additional materials and methods and tables containing descriptions of the *S. marcescens* strains and oligonucleotide primers used in this study. Download TEXT S1, DOCX file, 0.04 MB. Copyright © 2017 Anderson et al. 2017 Anderson et al. This content is distributed under the terms of the Creative Commons Attribution 4.0 International license.

### Construction of *S. marcescens* mutant strains.

Recombineering-based mutagenesis of *S. marcescens* was performed in accordance with the protocol of Thomason et al. (42). *S. marcescens* UMH9 harboring plasmid pSIM19 (43) was used as the parent strain, and the oligonucleotide primers used are listed in Text S1. For additional details and a description of the methods used for genetic complementation, see Text S1.

### Murine model of *S. marcescens* bacteremia.

*S. marcescens* strains were cultured overnight in LB medium and then subcultured to an OD at 600 nm (OD_600_) of 0.1 in LB and incubated at 37°C for 2 to 3 h. Bacteria were then collected by centrifugation and resuspended in an appropriate volume of phosphate-buffered saline (PBS). Female 6- to 8-week-old C57BL/6 mice were infected via tail vein injection with 0.1 ml of the bacterial suspension containing ~1 × 10^7^ CFU unless indicated otherwise. Mice were euthanized at 24 h or at the postinfection time indicated, and the spleen, liver, and kidneys were harvested in accordance with institution-approved protocols. The bacterial burden of infected mice was determined by plating serial dilutions of organ homogenates onto LB agar. For competition infections and bottleneck assessment experiments, wild-type bacteria were mixed with antibiotic-resistant mutant strains at the ratios indicated prior to infection. The density of each strain was determined for both the inoculum (input) and organ homogenates (output) by serial dilution and plating on LB containing or lacking antibiotics. The CI was calculated by determining the following ratio: (CFU_mutant_/CFU_wild type_)^output^/(CFU_mutant_/CFU_wild type_)^input^. Colonization bottleneck experiments are described in Text S1. All animal experiments were conducted by using protocols approved by the Institutional Animal Care and Use Committee.

### *In vivo* screen for *S. marcescens* fitness mutants.

Frozen stocks from five mutant pools, containing ~10,000 pSAM_Cm transconjugants each (see Text S1), were used to inoculate independent 20-ml LB cultures at a dilution of 1:100. Cultures were incubated for 2.5 h at 37°C before bacteria were collected by centrifugation and resuspended to a density of 1 × 10^8^ CFU/ml in PBS. Suspensions were used to infect four replicate C57BL/6 mice per mutant pool, for a total of 20 mice. Aliquots (1 ml) of each inoculum suspension were collected by centrifugation and stored at −80°C for subsequent isolation of genomic DNA to serve as the input samples. Two mice succumbed to infection prior to 24 h and were excluded from further analysis (pools 3 and 4, *n* = 3). The remaining mice were sacrificed, and spleens were homogenized in 1 ml of PBS. A 20-µl sample of each homogenate was removed for CFU determination, and the remaining volume was plated onto LB. The total bacterial growth recovered from each individual mouse was pooled to serve as the output samples. Bacteria from these pools were collected by centrifugation, and aliquots were frozen at −80°C for subsequent isolation of genomic DNA. The quantitative sequencing of transposon insertion sites and the identification of fitness genes and essential genes are described in Text S1.

### Microscopic examination of capsule production.

Capsule production by *S. marcescens* strains was assessed by negative staining with CR dye. Bacteria from LB plates incubated at 37°C were suspended in an aqueous solution of 1% CR and allowed to dry on the surface of a slide. As a control, bacteria were stained with a 1% solution of crystal violet for 2 min. Images were captured on an Olympus BX60 microscope with a 100× objective. To determine the size of capsule negative staining and of crystal violet-stained bacterial cells, images were converted to binary and quantitated with Fiji (44). Three fields per slide were captured, and the areas from ~300 cells per condition were plotted as a frequency distribution. Only cells that were well isolated were measured.

### Quantitation of extracellular uronic acids.

Extracellular uronic acids were measured on the basis of previously published methods (45–47) and extracted from overnight cultures of *S. marcescens* strains cultured in LB medium. For additional details, see Text S1.

### Growth of *S. marcescens* in magnesium-depleted medium.

The growth of the *mgtB* mutant in the presence of defined concentrations of magnesium was measured in M9 medium (48). The base medium contained 0.4% glucose, 0.1% Casamino acids, 0.1 mM CaCl_2_, 36 µM FeSO_4_, and 5 µg/ml thiamine. Magnesium-replete M9 was additionally supplemented with 1.0 mM MgSO_4_. Magnesium-depleted M9 medium was prepared by treatment of the base medium with Chelex 100 resin (Bio-Rad) prior to the addition of divalent cations. Following treatment, CaCl_2_ and FeSO_4_ were added along with MgSO_4_ at the concentrations indicated. *S. marcescens* strains were incubated in magnesium-replete M9 overnight, washed once, and then subcultured into magnesium-depleted M9 for 3 h. Strains were subcultured a second time in 100-well microplates containing magnesium-depleted M9 supplemented with different concentrations of magnesium. Growth was measured by OD_600_ determination every 15 min with a BioScreen C Analyzer at 37°C with continuous shaking.

### Growth and survival of *S. marcescens* in human serum.

The sensitivity of *S. marcescens* strains to serum bactericidal activity was tested by incubating bacteria in the presence of 10% normal human serum (Innovative Research). Bacteria were harvested from exponential-phase cultures by centrifugation and washed in PBS. Approximately 2 × 10^6^ CFU were added to serum diluted in PBS and were then incubated for 90 min at 37°C. Following incubation, bacterial suspensions were serially diluted and plated on LB agar for CFU determination. Serum HI by treatment at 56°C for 30 min was used as a negative control.

Growth of *S. marcescens* in human serum was tested with the M9 base medium described above supplemented with 20% HI serum. Overnight LB cultures of *S. marcescens* were harvested by centrifugation and washed once prior to the inoculation of 0.3-ml culture volumes into M9 basal medium supplemented with 20% HI human serum at an OD_600_ of 0.01. The OD_600_ of cultures was determined every 15 min with a BioScreen C Analyzer. Additional growth curves in M9 medium supplemented with 0.4% glucose and 0.1% Casamino acids were generated in the same manner. Bacterial aggregation in cultures containing 20% serum limited the accuracy of OD_600_ measurements, and thus, the density of viable bacteria over time was determined from independent 25-ml cultures grown under the same conditions.

## References

[1] Mahlen SD 2011 *Serratia* infections: from military experiments to current practice. Clin Microbiol Rev 24:755–791. doi:10.1128/CMR.00017-11. 21976608 PMC3194826

[2] Moradigaravand D, Boinett CJ, Martin V, Peacock SJ, Parkhill J 2016 Recent independent emergence of multiple multidrug-resistant *Serratia marcescens* clones within the United Kingdom and Ireland. Genome Res 26:1101–1109. doi:10.1101/gr.205245.116. 27432456 PMC4971767

[3] Iguchi A, Nagaya Y, Pradel E, Ooka T, Ogura Y, Katsura K, Kurokawa K, Oshima K, Hattori M, Parkhill J, Sebaihia M, Coulthurst SJ, Gotoh N, Thomson NR, Ewbank JJ, Hayashi T 2014 Genome evolution and plasticity of *Serratia marcescens*, an important multidrug-resistant nosocomial pathogen. Genome Biol Evol 6:2096–2110. doi:10.1093/gbe/evu160. 25070509 PMC4231636

[4] Haddy RI, Mann BL, Nadkarni DD, Cruz RF, Elshoff DJ, Buendia FC, Domers TA, Oberheu AM 1996 Nosocomial infection in the community hospital: severe infection due to *Serratia* species. J Fam Pract 42:273–277. 8636679

[5] Saito H, Elting L, Bodey GP, Berkey P 1989 *Serratia* bacteremia: review of 118 cases. Rev Infect Dis 11:912–920. doi:10.1093/clinids/11.6.912. 2602776

[6] Watanakunakorn C 1989 *Serratia* bacteremia: a review of 44 episodes. Scand J Infect Dis 21:477–483. doi:10.3109/00365548909037874. 2587951

[7] Braun V, Neuss B, Ruan Y, Schiebel E, Schöffler H, Jander G 1987 Identification of the *Serratia marcescens* hemolysin determinant by cloning into *Escherichia coli*. J Bacteriol 169:2113–2120. doi:10.1128/jb.169.5.2113-2120.1987. 2437098 PMC212107

[8] Hertle R, Hilger M, Weingardt-Kocher S, Walev I 1999 Cytotoxic action of *Serratia marcescens* hemolysin on human epithelial cells. Infect Immun 67:817–825. 9916096 10.1128/iai.67.2.817-825.1999 PMC96392

[9] González-Juarbe N, Mares CA, Hinojosa CA, Medina JL, Cantwell A, Dube PH, Orihuela CJ, Bergman MA 2015 Requirement for *Serratia marcescens* cytolysin in a murine model of hemorrhagic pneumonia. Infect Immun 83:614–624. doi:10.1128/IAI.01822-14. 25422267 PMC4294263

[10] Kurz CL, Chauvet S, Andrès E, Aurouze M, Vallet I, Michel GP, Uh M, Celli J, Filloux A, De Bentzmann S, Steinmetz I, Hoffmann JA, Finlay BB, Gorvel JP, Ferrandon D, Ewbank JJ 2003 Virulence factors of the human opportunistic pathogen *Serratia marcescens* identified by *in vivo* screening. EMBO J 22:1451–1460. doi:10.1093/emboj/cdg159. 12660152 PMC152903

[11] Shanks RMQ, Stella NA, Hunt KM, Brothers KM, Zhang L, Thibodeau PH 2015 Identification of SlpB, a cytotoxic protease from *Serratia marcescens*. Infect Immun 83:2907–2916. doi:10.1128/IAI.03096-14. 25939509 PMC4468537

[12] Marty KB, Williams CL, Guynn LJ, Benedik MJ, Blanke SR 2002 Characterization of a cytotoxic factor in culture filtrates of *Serratia marcescens*. Infect Immun 70:1121–1128. doi:10.1128/IAI.70.3.1121-1128.2002. 11854191 PMC127783

[13] Kamata R, Matsumoto K, Okamura R, Yamamoto T, Maeda H 1985 The serratial 56K protease as a major pathogenic factor in serratial keratitis. Clinical and experimental study. Ophthalmology 92:1452–1459. doi:10.1016/S0161-6420(85)33855-1. 3906492

[14] Shimuta K, Ohnishi M, Iyoda S, Gotoh N, Koizumi N, Watanabe H 2009 The hemolytic and cytolytic activities of *Serratia marcescens* phospholipase A (PhlA) depend on lysophospholipid production by PhlA. BMC Microbiol 9:261. doi:10.1186/1471-2180-9-261. 20003541 PMC2800117

[15] Létoffé S, Ghigo JM, Wandersman C 1994 Iron acquisition from heme and hemoglobin by a *Serratia marcescens* extracellular protein. Proc Natl Acad Sci U S A 91:9876–9880. doi:10.1073/pnas.91.21.9876. 7937909 PMC44920

[16] Flyg C, Kenne K, Boman HG 1980 Insect pathogenic properties of *Serratia marcescens*: phage-resistant mutants with a decreased resistance to *Cecropia* immunity and a decreased virulence to *Drosophila*. J Gen Microbiol 120:173–181. doi:10.1099/00221287-120-1-173. 7012273

[17] Merino S, Camprubí S, Albertí S, Benedí VJ, Tomás JM 1992 Mechanisms of *Klebsiella pneumoniae* resistance to complement-mediated killing. Infect Immun 60:2529–2535. 1587619 10.1128/iai.60.6.2529-2535.1992 PMC257192

[18] Whitfield C 2006 Biosynthesis and assembly of capsular polysaccharides in *Escherichia coli*. Annu Rev Biochem 75:39–68. doi:10.1146/annurev.biochem.75.103004.142545. 16756484

[19] Aucken HM, Wilkinson SG, Pitt TL 1997 Identification of capsular antigens in *Serratia marcescens*. J Clin Microbiol 35:59–63. 8968881 10.1128/jcm.35.1.59-63.1997 PMC229512

[20] Aucken HM, Wilkinson SG, Pitt TL 1998 Re-evaluation of the serotypes of *Serratia marcescens* and separation into two schemes based on lipopolysaccharide (O) and capsular polysaccharide (K) antigens. Microbiology 144:639–653. doi:10.1099/00221287-144-3-639. 9534235

[21] Bachman MA, Breen P, Deornellas V, Mu Q, Zhao L, Wu W, Cavalcoli JD, Mobley HLT 2015 Genome-wide identification of *Klebsiella pneumoniae* fitness genes during lung infection. mBio 6:e00775. doi:10.1128/mBio.00775-15. 26060277 PMC4462621

[22] Goodman AL, Wu M, Gordon JI 2011 Identifying microbial fitness determinants by insertion sequencing using genome-wide transposon mutant libraries. Nat Protoc 6:1969–1980. doi:10.1038/nprot.2011.417. 22094732 PMC3310428

[23] Zomer A, Burghout P, Bootsma HJ, Hermans PWM, van Hijum SA 2012 Essentials: software for rapid analysis of high throughput transposon insertion sequencing data. PLoS One 7:e43012. doi:10.1371/journal.pone.0043012. 22900082 PMC3416827

[24] Liu D, Cole RA, Reeves PR 1996 An O-antigen processing function for Wzx (RfbX): a promising candidate for O-unit flippase. J Bacteriol 178:2102–2107. doi:10.1128/jb.178.7.2102-2107.1996. 8606190 PMC177911

[25] Islam ST, Lam JS 2013 Wzx flippase-mediated membrane translocation of sugar polymer precursors in bacteria. Environ Microbiol 15:1001–1015. doi:10.1111/j.1462-2920.2012.02890.x. 23016929

[26] Rahn A, Whitfield C 2003 Transcriptional organization and regulation of the *Escherichia coli* K30 group 1 capsule biosynthesis (cps) gene cluster. Mol Microbiol 47:1045–1060. doi:10.1046/j.1365-2958.2003.03354.x. 12581358

[27] Bailey MJ, Hughes C, Koronakis V 1997 RfaH and the *ops* element, components of a novel system controlling bacterial transcription elongation. Mol Microbiol 26:845–851. doi:10.1046/j.1365-2958.1997.6432014.x. 9426123

[28] Pan YJ, Lin TL, Chen CT, Chen YY, Hsieh PF, Hsu CR, Wu MC, Wang JT 2015 Genetic analysis of capsular polysaccharide synthesis gene clusters in 79 capsular types of *Klebsiella* spp. Sci Rep 5:15573. doi:10.1038/srep15573. 26493302 PMC4616057

[29] Weissborn AC, Liu Q, Rumley MK, Kennedy EP 1994 UTP: alpha-d-glucose-1-phosphate uridylyltransferase of *Escherichia coli*: isolation and DNA sequence of the *galU* gene and purification of the enzyme. J Bacteriol 176:2611–2618. doi:10.1128/jb.176.9.2611-2618.1994. 8169209 PMC205399

[30] Groisman EA, Hollands K, Kriner MA, Lee EJ, Park SY, Pontes MH 2013 Bacterial Mg^2+^ homeostasis, transport, and virulence. Annu Rev Genet 47:625–646. doi:10.1146/annurev-genet-051313-051025. 24079267 PMC4059682

[31] Kazmierczak KM, Rabine S, Hackel M, McLaughlin RE, Biedenbach DJ, Bouchillon SK, Sahm DF, Bradford PA 2016 Multiyear, multinational survey of the incidence and global distribution of metallo-β-lactamase-producing *Enterobacteriaceae* and *Pseudomonas aeruginosa*. Antimicrob Agents Chemother 60:1067–1078. doi:10.1128/AAC.02379-15. 26643349 PMC4750703

[32] McKenna M 2013 Antibiotic resistance: the last resort. Nature 499:394–396. doi:10.1038/499394a. 23887414

[33] Lin CS, Horng JT, Yang CH, Tsai YH, Su LH, Wei CF, Chen CC, Hsieh SC, Lu CC, Lai HC 2010 RssAB-FlhDC-ShlBA as a major pathogenesis pathway in *Serratia marcescens*. Infect Immun 78:4870–4881. doi:10.1128/IAI.00661-10. 20713626 PMC2976324

[34] Majdalani N, Gottesman S 2005 The Rcs phosphorelay: a complex signal transduction system. Annu Rev Microbiol 59:379–405. doi:10.1146/annurev.micro.59.050405.101230. 16153174

[35] Clarke DJ 2010 The Rcs phosphorelay: more than just a two-component pathway. Future Microbiol 5:1173–1184. doi:10.2217/fmb.10.83. 20722597

[36] Gottesman S, Trisler P, Torres-Cabassa A 1985 Regulation of capsular polysaccharide synthesis in *Escherichia coli* K-12: characterization of three regulatory genes. J Bacteriol 162:1111–1119. 3888955 10.1128/jb.162.3.1111-1119.1985 PMC215891

[37] Di Venanzio G, Stepanenko TM, García Véscovi E 2014 *Serratia marcescens* ShlA pore-forming toxin is responsible for early induction of autophagy in host cells and is transcriptionally regulated by RcsB. Infect Immun 82:3542–3554. doi:10.1128/IAI.01682-14. 24914224 PMC4187834

[38] Rahn A, Beis K, Naismith JH, Whitfield C 2003 A novel outer membrane protein, Wzi, is involved in surface assembly of the *Escherichia coli* K30 group 1 capsule. J Bacteriol 185:5882–5890. doi:10.1128/JB.185.19.5882-5890.2003. 13129961 PMC193962

[39] Bushell SR, Mainprize IL, Wear MA, Lou H, Whitfield C, Naismith JH 2013 Wzi is an outer membrane lectin that underpins group 1 capsule assembly in *Escherichia coli*. Structure 21:844–853. doi:10.1016/j.str.2013.03.010. 23623732 PMC3791409

[40] Marchesi JR, Sato T, Weightman AJ, Martin TA, Fry JC, Hiom SJ, Dymock D, Wade WG 1998 Design and evaluation of useful bacterium-specific PCR primers that amplify genes coding for bacterial 16S rRNA. Appl Environ Microbiol 64:795–799. 9464425 10.1128/aem.64.2.795-799.1998 PMC106123

[41] Bertani G 1951 Studies on lysogenesis. I. The mode of phage liberation by lysogenic Escherichia coli. J Bacteriol 62:293–300. 14888646 10.1128/jb.62.3.293-300.1951 PMC386127

[42] Thomason LC, Sawitzke JA, Li X, Costantino N, Court DL 2014 Recombineering: genetic engineering in bacteria using homologous recombination. Curr Protoc Mol Biol 106:1.16.1-39. doi:10.1002/0471142727.mb0116s106. 24733238

[43] Datta S, Costantino N, Court DL 2006 A set of recombineering plasmids for Gram-negative bacteria. Gene 379:109–115. doi:10.1016/j.gene.2006.04.018. 16750601

[44] Schindelin J, Arganda-Carreras I, Frise E, Kaynig V, Longair M, Pietzsch T, Preibisch S, Rueden C, Saalfeld S, Schmid B, Tinevez JY, White DJ, Hartenstein V, Eliceiri K, Tomancak P, Cardona A 2012 Fiji: an open-source platform for biological-image analysis. Nat Methods 9:676–682. doi:10.1038/nmeth.2019. 22743772 PMC3855844

[45] Blumenkrantz N, Asboe-Hansen G 1973 New method for quantitative determination of uronic acids. Anal Biochem 54:484–489. doi:10.1016/0003-2697(73)90377-1. 4269305

[46] Domenico P, Diedrich DL, Cunha BA 1989 Quantitative extraction and purification of exopolysaccharides from *Klebsiella pneumoniae*. J Microbiol Methods 9:211–219. doi:10.1016/0167-7012(89)90038-9.

[47] Favre-Bonte S, Joly B, Forestier C 1999 Consequences of reduction of *Klebsiella pneumoniae* capsule expression on interactions of this bacterium with epithelial cells. Infect Immun 67:554–561. 9916058 10.1128/iai.67.2.554-561.1999 PMC96354

[48] Miller JH 1972 Experiments in molecular genetics. Cold Spring Harbor Laboratory, Cold Spring Harbor, NY.

