# Supplementary material for: Capsule Production and Glucose Metabolism Dictate Fitness during Serratia marcescens Bacteremia
Source: mBio. 2017 May 23;8(3):e00740-17. doi: 10.1128/mBio.00740-17 (PMC5442460; doi:10.1128/mBio.00740-17)
Supplement: TEXT S1 [file mbo003173325s1.docx]

**TEXT S1**

**Supplemental Materials and Methods**

***In vitro* growth of *S. marcescens* mutants**

The growth rate of all engineered *S. marcescens* mutant strains was determined *in vitro* and compared with parent strain UMH9. Strains were cultured in LB medium overnight at 37°C followed by subculture into LB at OD_600_ = 0.01. Suspensions were inoculated into 100-well microplates in a volume of 0.3 ml and incubated in a BioScreen C Analyzer at 37°C with continuous shaking. The optical density of cultures was measured every 15 minutes.

**Genome sequencing, assembly, and annotation**

Genomic DNA was isolated from *S. marcescens* strains using Qiagen 100/G genomic-tips according to the manufacturer’s recommendations. Sequencing was performed by the University of Michigan DNA Sequencing Core via the PacBio RS II platform. Sequence reads were assembled using the PacBio SMRT analysis portal by the University of Michigan Bioinformatics Core. Initial assemblies were trimmed with the aid of the Gepard alignment program (1) to identify repetitive end sequences. Contigs with ambiguous placement in the genome were resolved by PCR and further sequencing. All plasmid sequences identified during the assembly process were verified via PCR and additional sequencing. Nucleotide position number one was assigned for each assembly based on the *E. coli* numbering convention. Preliminary genome annotation was performed using the Rapid Annotation using Subsystem Technology (RAST) server (2). Sequences were annotated via PGAP upon NCBI deposition and accession numbers are listed in Table S1.

**Bioinformatic analysis of the capsule biosynthesis genetic locus**

The chromosomal region encoding capsule biosynthesis genes was manually annotated using information generated from BLAST searches to predict appropriate start and stop codons for each ORF. The similarity of the UMH9 locus to capsule loci from other bacterial species was also determined using BLAST. The capsule loci from the 11 different *S. marcescens* bloodstream infection isolates that were sequenced in this study were aligned in MegAlign Pro (DNAstar) using the Clustal Omega algorithm (3). Each chromosomal fragment used in the multisequence alignment was bounded by conserved genes; namely, *galU* encoding a UTP--glucose-1-phosphate uridylytransferase and *lipB* encoding the ATP-binding component of an ABC exporter, that latter of which is not involved in capsule production.

**Assay for colonization bottleneck during murine infection**

Colonization bottlenecks were assessed by competition infections using strains UMH9 and UMH9^Nal^. Both strains were cultivated and prepared as described above, then mixed in 1:1:000, 1:5,000, and 1:10,000 ratios (UMH9^Nal^:UMH9) prior to infection. Mice were sacrificed at 24 hrs post-infection and the spleens were harvested and homogenized in PBS. Due to low numbers of UMH9^Nal^ at the lowest tested ratios, it was necessary to expand the entire population prior to plating by mixing 2 ml of spleen homogenate with 3 ml of LB and incubating for 5 hrs at 37 °C. Suspensions were then serially diluted and colony counts were determined on LB and LB containing nalidixic acid to determine the CI as described above. Spleen homogenates from the 1:1 ratio were also serially diluted and plated directly without expansion in LB as a control. The relative fitness of the two strains *in vitro* was determined by 8-hr co-culture in LB medium at ratios of 1:100 and 1:1,000 (UMH9^Nal^:UMH9).

**Construction of *S. marcescens* mutant strains**

Recombineering-based mutagenesis of *S. marcescens* was performed according to the protocol of Thomason *et al*. (4). *S. marcescens* UMH9 harboring plasmid pSIM19 (5) was used as the parent strain. The kanamycin resistance gene from plasmid pKD4 (6) was PCR-amplified using extended oligonucleotides having ~50-bp of sequence on the 5ʹ ends homologous to the targeted *S. marcescens* chromosomal sequence (Table S2). UMH9(pSIM19) was treated for 20 min at 42°C to allow for expression of λ Red recombineering functions prior to preparation of competent bacteria. Bacteria were then transformed with ~1 µg of DpnI-treated PCR product via electroporation and recombinants were selected on LB containing kanamycin. The presence of the desired mutation was confirmed by PCR and sequencing. Plasmid pSIM19 was cured from all mutant strains prior to phenotypic analysis using established protocols (4). All engineered *S. marcescens* mutants contained internal in-frame deletions and *nptII* kanamycin resistance gene insertions as described in Table S1.

**Genetic complementation**

For genetic complementation of the *wzx*, *pgm*, and *mgtB* mutations, DNA fragments containing the respective open reading frames were PCR-amplified using the primers listed in Table S2. The resulting PCR products were incorporated into the pCR^TM^4Blunt-TOPO^®^ plasmid (ThermoFisher) according to the manufacturer’s instructions and transformed into electrocompetent *E. coli* TOP10 cells (ThermoFisher). Insert fragments were then liberated from recombinant pCR^TM^4Blunt-TOPO^®^ plasmids by restriction digest (*wzx*, PstI and BamHI; *pgm*, EcoRI and HindIII; *mgtB*, EcoRI and HindIII) and ligated to appropriately-digested pBBR1MCS-5 (7), followed by electroporation of TOP10 cells. Recombinant pBBR1MCS-5 plasmids were identified by restriction digest and verified by DNA sequencing (University of Michigan Sequencing Core). *S. marcescens* mutant strains were treated at 42°C for 20 minutes prior to preparation of competent bacteria and electroporation with recombinant pBBR1MCS-5 derivatives or the empty vector. *S. marcescens* transformants were selected on LB containing gentamicin.

**Transposon insertion mutagenesis**

Transposon insertion mutants were obtained by conjugative transfer of pSAM_Cam into *S. marcescens* strain UMH9 using *E. coli* S17-1 as the donor (8). Suicide plasmid pSAM_Cam (9) encodes a copy of the *mariner*-based transposon with MmeI restriction sites in the terminal repeats and is a derivative of pSAM-Ec (10, 11). Recipient cells grown to mid-log phase were incubated at 42°C for 20 min prior to mixing with donor bacteria in a 1:1 ratio. Mating was allowed to occur for 6 hrs at 37°C followed by plating on LB agar containing kanamycin and ampicillin to select for transconjugants with integrated transposons. Kanamycin- and ampicillin-resistant transconjugants from primary selection plates were combined in pools of ~10,000 CFU and stored at -80°C for use in the INSeq screen. To assess whether transposon integration occurred randomly in the UMH9 chromosome, genomic DNA from 47 individual isolates was purified (12) and digested with SalI for Southern blot analysis. A DIG-labeled probe that hybridizes to an 501-bp internal portion of the transposon-encoded kanamycin resistance gene (10) was prepared to according the manufacturer’s recommendations and used to compare the transposon integration site between isolates.

**Identification of fitness genes by INSeq**

Purification of genomic DNA from input and output pools was performed as described previously (12). PCR amplification of transposon insertion site sequences was performed based on the method of Goodman *et al*., including the use of previously described oligonucleotide sequences (13). Briefly, genomic DNA (1 µg) isolated from input and output pools was used as the template for linear amplification with primer BioSamA and Platinum Pfx polymerase (ThermoFisher). Second strand synthesis was performed using Klenow (exo^-^) enzyme and random hexanucleotides followed by MmeI digestion of double-stranded products. Double-stranded sequencing adapters (LIB_Adapt) containing four-nucleotide barcodes were then ligated to MmeI-digested DNA. Input pools were processed as technical duplicates, with each replicate ligated to an adapter having a unique barcode. Output samples from individual mice were also assigned unique barcodes. PCR amplification of the ligated DNA fragments was performed using primers LIB-PCR5 and LIB-PCR3 and the resulting products were purified by gel extraction prior to sequencing. Sufficient product for sequencing could not be obtained from one output sample, which was eliminated from further analysis (pool 5, n=3). Purified PCR products containing transposon-chromosome DNA junctions were pooled for multiplexing and sequenced on the Illumina HiSeq platform using 50 cycle single-end reads by the University of Michigan DNA Sequencing Core Facility. Sequences were deposited in the NCBI Sequence Read Archive (SRP095178).

Sequence reads were mapped to the UMH9 annotated genome using the ESSENTIALS pipeline (14). User-defined parameters for the ESSENTIALS analysis employed the default settings with the following exceptions: library size was set to 50,000, zero barcode mismatches were allowed, and the Cox-Reid method of dispersion estimation was used in EdgeR. Both essential and conditionally essential fitness genes reported herein were identified based on the statistical cutoff of fold-change > 2.0 and adjusted P < 0.05.

The TnseqDiff function in the Tnseq R package (L. Zhao, M. T. Anderson, W. Wu, Y. Li, H. L. T. Mobley, and M. A. Bachman, submitted for publication) was also used to identify fitness genes from the same data set. TnseqDiff utilizes two steps to estimate the fitness contribution for each gene. First, a CD function (15) is constructed to collect fitness evidence for each insertion by comparing read counts in the input sample to the output samples. Second, the insertion-level CD functions are combined to infer the fitness effect for the corresponding gene. The first step relies on the limma package (16). Specifically, read counts were normalized to account for the differences in library sizes, then were transformed to log-counts per million and modelled as a linear function of the condition (input/output) at each insertion site. To consider the over-dispersion of the count data produced from the sequencing technique, a precision weight was estimated for each observation from the mean-variance relationship of the log-counts and was then entered into the linear modeling (17). The CD function was constructed using the slope (*i*.*e*., log fold change) estimates (mean, standard error and degrees of freedom) from the linear model. In the second step, the insertion-level CD functions (15) are combined to obtain a single CD function for the corresponding gene and then derived a P-value from the combined CD function to infer the fitness effect of that gene. These P-values were further adjusted for multiple hypothesis correction (18). Significant fitness genes were considered to have a fold-change > 2.0 and adjusted P < 0.05. The Tnseq R package can be installed from the Comprehensive R Archive Network (CRAN) (https://cran.r-project.org).

**Quantitation of extracellular uronic acids.**

*S. marcescens* strains were cultured overnight (37 °C) in 4 ml of LB medium, collected by centrifugation, and resuspended in 1 ml of PBS. A 0.5 ml volume of each suspension was mixed with 0.1 ml Zwittergent 3-14 in 100 mM citric acid buffer pH 2.0 and incubated at 50 °C for 20 minutes. Bacterial cells were pellet by centrifugation and 0.25 ml of cell-free supernatant was mixed with 1 ml absolute ethanol and incubated on ice for 20 minutes. The carbohydrate-containing precipitate was collected by centrifugation and rehydrated in 0.2 ml water, followed by addition of 1 ml of 12.5 mM sodium tetraborate in concentrated sulfuric acid. The solution was incubated at 100 °C for five minutes then cooled to room temperature. Uronic acids were detected by addition of 20 µl of 0.15% 3-hydroxydiphenyl in 0.5% sodium hydroxide followed by measurement of absorbance at 520 nm. A duplicate sample from each strain was prepared as described above, but treated with 0.5% sodium hydroxide alone and used to measure the background A_520_. The level of uronic acids in background-subtracted values was determined based on a standard curve generated with glucuronic acid. All values were normalized to the optical density of each culture.

**References**

1. **Krumsiek J, Arnold R, Rattei T.** 2007. Gepard: a rapid and sensitive tool for creating dotplots on genome scale. Bioinformatics **23:**1026-1028.

2. **Overbeek R, Olson R, Pusch GD, Olsen GJ, Davis JJ, Disz T, Edwards RA, Gerdes S, Parrello B, Shukla M, Vonstein V, Wattam AR, Xia F, Stevens R.** 2014. The SEED and the Rapid Annotation of microbial genomes using Subsystems Technology (RAST). Nucleic Acids Res **42:**D206-214.

3. **Sievers F, Wilm A, Dineen D, Gibson TJ, Karplus K, Li W, Lopez R, McWilliam H, Remmert M, Söding J, Thompson JD, Higgins DG.** 2011. Fast, scalable generation of high‐quality protein multiple sequence alignments using Clustal Omega. Mol Syst Biol **7:**539.

4. **Thomason LC, Sawitzke JA, Li X, Costantino N, Court DL.** 2014. Recombineering: genetic engineering in bacteria using homologous recombination. Curr Protoc Mol Biol **106:**1 16 11-39.

5. **Datta S, Costantino N, Court DL.** 2006. A set of recombineering plasmids for Gram-negative bacteria. Gene **379:**109-115.

6. **Datsenko KA, Wanner BL.** 2000. One-step inactivation of chromosomal genes in *Escherichia coli* K-12 using PCR products. Proc Nat Acad Sci U S A **97:**6640-6645.

7. **Kovach ME, Elzer PH, Hill DS, Robertson GT, Farris MA, Roop RM, 2nd, Peterson KM.** 1995. Four new derivatives of the broad-host-range cloning vector pBBR1MCS, carrying different antibiotic-resistance cassettes. Gene **166:**175-176.

8. **Simon R, Priefer U, Puhler A.** 1983. A broad host range mobilization system for *in vivo* genetic-engineering - transposon mutagenesis in Gram-negative bacteria. Bio-technology **1:**784-791.

9. **Bachman MA, Breen P, Deornellas V, Mu Q, Zhao L, Wu W, Cavalcoli JD, Mobley HLT.** 2015. Genome-wide identification of *Klebsiella pneumoniae* fitness genes during lung infection. mBio **6:**e00775.

10. **Wiles TJ, Norton JP, Russell CW, Dalley BK, Fischer KF, Mulvey MA.** 2013. Combining quantitative genetic footprinting and trait enrichment analysis to identify fitness determinants of a bacterial pathogen. PLoS Genet **9:**e1003716.

11. **Goodman AL, McNulty NP, Zhao Y, Leip D, Mitra RD, Lozupone CA, Knight R, Gordon JI.** 2009. Identifying genetic determinants needed to establish a human gut symbiont in its habitat. Cell Host Microbe **6:**279-289.

12. **Wilson K.** 2001. Preparation of genomic DNA from bacteria. Curr Protoc Mol Biol **56:**2.4.1-2.4.5

13. **Goodman AL, Wu M, Gordon JI.** 2011. Identifying microbial fitness determinants by insertion sequencing using genome-wide transposon mutant libraries. Nat Protoc **6:**1969-1980.

14. **Zomer A, Burghout P, Bootsma HJ, Hermans PWM, Hijum SAFTv.** 2012. ESSENTIALS: software for rapid analysis of high throughput transposon insertion sequencing data. PLoS ONE **7:**e43012.

15. **Singh K, Xie M, Strawderman WE.** 2005. Combining information from independent sources through confidence distributions. Ann Stat **33:**159-183.

16. **Ritchie ME, Phipson B, Wu D, Hu Y, Law CW, Shi W, Smyth GK.** 2015. Limma powers differential expression analyses for RNA-sequencing and microarray studies. Nucleic Acids Res **43:**e47.

17. **Smyth GK.** 2004. Linear models and empirical bayes methods for assessing differential expression in microarray experiments. Stat Appl Genet Mol Biol **3:**1–25.

18. **Storey JD, Tibshirani R.** 2003. Statistical significance for genomewide studies. Proc Nat Acad Sci U S A **100:**9440-9445.

**Table S1. *S. marcescens* strains used in this study.**

| **Strain name** | **Relevant genotype** | **Description** |
| --- | --- | --- |
| UMH1 | WT^a^ | bloodstream infection isolate, CP018915 |
| UMH2 | WT | bloodstream infection isolate, CP018924 |
| UMH3 | WT | bloodstream infection isolate, CP018925 |
| UMH5 | WT | bloodstream infection isolate, CP018917 |
| UMH6 | WT | bloodstream infection isolate, CP018926 |
| UMH7 | WT | bloodstream infection isolate, CP018919 |
| UMH8 | WT | bloodstream infection isolate, CP018927 |
| UMH9 | WT | bloodstream infection isolate, CP018923 |
| UMH10 | WT | bloodstream infection isolate, CP018928 |
| UMH11 | WT | bloodstream infection isolate, CP018929 |
| UMH12 | WT | bloodstream infection isolate, CP018930 |
| UMH9^Nal^ | ND^b^ | Spontaneous nalidixic acid-resistant derivative of UMH9 |
| *wzx* | Δ*wzx*::*nptII* | deletion of a 996-bp internal fragment of UMH9_0919 and insertion of 1496-bp *nptII*-containing fragment from pKD4 |
| UMH9_0939 | ΔUMH9_0939::*nptII* | deletion of a 714-bp internal fragment of UMH9_0939 and insertion of 1496-bp *nptII*-containing fragment from pKD4 |
| *mgtB* | Δ*mgtB*::*nptII* | deletion of a 2172-bp internal fragment of UMH9_2349 and insertion of 1496-bp *nptII*-containing fragment from pKD4 |
| *rcsB* | Δ*rcsB*::*nptII* | deletion of a 447-bp internal fragment of UMH9_2631 and insertion of 1496-bp *nptII*-containing fragment from pKD4 |
| UMH9_0544 | ΔUMH9_0544::*nptII* | deletion of a 219-bp internal fragment of UMH9_0544 and insertion of 1476-bp *nptII*-containing fragment from pKD4 |
| *pgm* | Δ*pgm*::*nptII* | deletion of a 1611-bp internal fragment of UMH9_0547 and insertion of 1476-bp *nptII*-containing fragment from pKD4 |
| *pfkA* | Δ*pfkA*::*nptII* | deletion of a 933-bp internal fragment of UMH9_4007 and insertion of 1476-bp *nptII*-containing fragment from pKD4 |

a, wild-type

b, not determined**Table S2. Oligonucleotide primers used for recombineering and cloning.**

| **Primer name** | **Sequence (5ʹ-3ʹ)** |
| --- | --- |
| *recombineering* |  |
| Δ*wzx*::kmF | actctttatttgtccgttattcagatcttgatcattttcatttcgttcagtgtaggctggagctgcttc |
| Δ*wzx*::kmR | gtcgcttgccacaataaaaagtcactgcacatgcataaaaactggatgggaattagccatggtcc |
| Δ0939::kmF | tgatctggttattgcgacttggtggcgtactgtattttatctcaacaaagtgtaggctggagctgcttc |
| Δ0939::kmR | taataactcccgatgcaataagctgctcgcacgctgctgaaaactctttatgggaattagccatggtcc |
| Δ*mgtB*::kmF | aggcgccgcatgcctttatccagctgatcaaggctttcaacaacccgtttgtgtaggctggagctgcttc |
| Δ*mgtB*::kmR | atatgcaccaccagcgtttgcgacagcagcccctcaatgaaccagccggaatgggaattagccatggtcc |
| Δ*rcsB*::kmF | atgaataacctgaacgtaattattgctgatgaccatcctatcgtactgttgtgtaggctggagctgcttc |
| Δ*rcsB*::kmR | tgatggttttgatgctgcggttgagcttcttggcgatctcggtcaccaggatgggaattagccatggtcc |
| Δ0544::kmF | gatgcaaactcccttgcagtaccgctacctatggcaaaagaacaaacggatgtaggctggagctgcttcg |
| Δ0544::kmR | tcaatgttccggctgttcttccgccagttgcgccaacagcatctgctcgcatatgaatatcctccttagt |
| Δ*pgm*::kmF | attgcccggtcaccgacagggatgagggagatatgtcgatggcgaataattgtaggctggagctgcttcg |
| Δ*pgm*::kmR | cagcgctttttatcaatcagggaaagtgattacttggcggaagccagcaccatatgaatatcctccttagt |
| Δ*pfkA*::kmR | agaaattgcatctacacagttcagaggtagtcatgatcaagaaaatcggttgtaggctggagctgcttcg |
| Δ*pfkA*::kmF | gcaagcggaggctcttttgtcgtgcggtgagcggcgtatcagtacagtttcatatgaatatcctccttagt |
| *cloning* |  |
| *wzx*FOR | ctgcagctggcatgcgcttaacttcctg |
| *wzx*REV | ggatccgtaacaccgccctttccaacca |
| *pgm*FOR | aagcttggccgtacccgcacctatttc |
| *pgm*REV | gaattcgacggcggccctcttcacat |
| *mgtB*FOR | aagcttcgccggccgcacaaaaagaaat |
| *mgtB*REV | gaattctagcggagcgggggcgaagaat |
